# BBNSF: Blockchain-Based Novel Secure Framework Using RP^2^-RSA and ASR-ANN Technique for IoT Enabled Healthcare Systems

**DOI:** 10.3390/s22239448

**Published:** 2022-12-02

**Authors:** Mohit Kumar, Priya Mukherjee, Sahil Verma, Kavita, Maninder Kaur, S. Singh, Martyna Kobielnik, Marcin Woźniak, Jana Shafi, Muhammad Fazal Ijaz

**Affiliations:** 1 Department of Information Technology, MIT-ADT University, Pune 412201, India; 2 RBSPL, Bangalore 560008, India; 3 Faculty of Computer Science and Engineering, SGT University, Gurugram 122505, India; 4 Department of Computer Science and Applications, Guru Gobind Singh College for Women, Chandigarh 160019, India; 5 Division of Research & Innovation, Uttaranchal University, Dehradun 248007, India; 6 Faculty of Applied Mathematics, Silesian University of Technology, Kaszubska 23, 44-100 Gliwice, Poland; 7 Department of Computer Science, College of Arts and Science, Prince Sattam bin Abdul Aziz University, Wadi Ad-Dawasir 11991, Saudi Arabia; 8 Department of Intelligent Mechatronics Engineering, Sejong University, Seoul 05006, Republic of Korea

**Keywords:** Internet of Medical Things (IoMT), data privacy, blockchain, security, reversed public-private keys combined Rivest–Shamir–Adleman, correlation factor-induced salp swarm optimization algorithm, data encryption, classification

## Abstract

The wearable healthcare equipment is primarily designed to alert patients of any specific health conditions or to act as a useful tool for treatment or follow-up. With the growth of technologies and connectivity, the security of these devices has become a growing concern. The lack of security awareness amongst novice users and the risk of several intermediary attacks for accessing health information severely endangers the use of IoT-enabled healthcare systems. In this paper, a blockchain-based secure data storage system is proposed along with a user authentication and health status prediction system. Firstly, this work utilizes reversed public-private keys combined Rivest–Shamir–Adleman (RP^2^-RSA) algorithm for providing security. Secondly, feature selection is completed by employing the correlation factor-induced salp swarm optimization algorithm (CF-SSOA). Finally, health status classification is performed using advanced weight initialization adapted SignReLU activation function-based artificial neural network (ASR-ANN) which classifies the status as normal and abnormal. Meanwhile, the abnormal measures are stored in the corresponding patient blockchain. Here, blockchain technology is used to store medical data securely for further analysis. The proposed model has achieved an accuracy of 95.893% and is validated by comparing it with other baseline techniques. On the security front, the proposed RP^2^-RSA attains a 96.123% security level.

## 1. Introduction

The change in life expectancy in developed countries has led to radical changes in the healthcare industry [1]. Specialized devices called wearable health devices that continuously monitor the real-time body conditions of the patient have gained immense popularity [2]. Since it can be personalized and can be used for continuous monitoring of vital parameters of a person it has become one of the most propitious platforms [3]. To frequently check the actions of a person without any disturbance, wearable devices are worn by an individual [4]. IoMT devices are normally affixed to the human body for collecting numerous types of sensitive data [5]. IoMT is utilized for removing medical errors and improving work productivity [6]. It consists of medical devices that are interconnected and can share data securely [7]. This shared data contains critical information about patients and by utilizing this information doctors can advise patients accordingly [8]. To handle diseases and drugs, to ameliorate the treatment methods together with the patient’s experience, and to lessen the cost and errors, IoMT solutions are used by healthcare providers [9]. The safety and confidentiality of IoMT-based healthcare systems have acquired inadequate attention because of the fast development of IoMT [10,11,12]. There is a possibility of breaching the patient’s personal and biological data, so the safety of this data is more significant [13,14,15,16]. For instance, if the smart medical device is attacked by an invader remotely, then the invader can hurt the life of the patient (i.e., with the help of the smart pacemaker, the invader could easily attack the patient’s life, which may also lead to death) [17]. Leakage of sensitive data is undoubtedly dangerous to the health of the patient, so ensuring the privacy of data has become one of the main challenges in IoMT [18]. One promising solution to the challenges mentioned above is to integrate blockchain with IoMT [19,20]. In the blockchain, data are deposited in a block, and each block points to the previous block’s hash and adopts symmetric encryption to maintain security [21,22,23,24]. Therefore, the data cannot be changed, and accessing data from the network by different parties can only be completed in accordance with stakeholders’ policy [25,26]. Since an invader can retrieve the patient’s health state with the aid of the doctor’s identity, the confidentiality of the patient’s information is safeguarded with the support of content privacy which protects the data from any leakage [27,28,29]. Thus, for strong security guarantees in an integrated healthcare environment user authentication is a very important step. Blockchains are transformative technology that has the potential to change many sectors [30,31,32]. On the other hand, the downside of Blockchain technology is that it consumes a lot of energy to remain operational because they are incentivized to solve complex mathematical problems. The authentication and storage schemes used in IoMT are centralized in nature; hence, the prevailing security techniques in addition to privacy techniques are not suitable for IoMT devices. Therefore, the proposed work encompasses a BC-centered secure data storage system together with user authentication and HS prediction method. In this paper, we have proposed a novel secure framework for IoMT. For storing medical data securely blockchain was utilized. The proposed work employs RP^2^RSA for encrypting and decrypting the data. The feature selection is performed using CF-SOA. Additionally, the health status classification is completed using advanced weight initialization adapted SignReLU activation function-based artificial neural network (ASR-ANN) which classifies the status as normal and abnormal. The remainder of the paper is outlined as follows: Section 2 describes some of the existing methodologies related to the proposed work. A detailed description of the proposed framework is mentioned in Section 3. In Section 4, the result of the proposed model and its comparisons are presented. At last, in Section 5, the paper is concluded along with future work.

## 2. Background and Related Work

This section presents a comprehensive discussion of related work on different techniques used by the researchers over the years. The chosen publications provide an overview of many approaches, classification algorithms, role of blockchain schemes in IoMT and different optimization strategies that have been utilized by different researchers in recent years. Randhir Kumar and Rakesh Tripathi [33] created the concept which provided safety along with confidentiality of IoMT through a smart contract-enabled consortium blockchain (BC) network. The two levels of the framework were the initialization level and the authentication level. The smart contract-enabled interplanetary file systems (IPFS) cluster node recorded the patient and the patient’s particular devices, at the initialization level. Then, the patient ID (PID) and device ID (DID) were distributed into the BC network. The mapping of patient ID (PID), device ID (DID), in addition to the device public address (DIP) was incorporated in the authentication level, and after that, it was distributed into the BC network of IoMT. From the perspective of safety and confidentiality of device-generated medical data, the experimental outcomes confirmed that the model was effective. IPFS resulted in low latency and created complicated peer-to-peer connections. Samira Akhbarifar et al. [34] provisioned safety for health along with medical data in a cloud-based environment by establishing a remote health monitoring model, which used a method of lightweight encryption. To convey the medical data safely to the service providers of communication, lightweight data encryption was performed. Through classification steps, the data were pre-processed and evaluated. The finest outcome amongst the RF MLP, SVM, and J48 classifiers for 10-fold cross-validation using the K-star classification method with an accuracy of 95%, precision of 94.5%, recall of 93.5, and 93.99% f-score was obtained from the experimental results. However, it didn’t consider the requisites in key-dependent S-Box designing to give more security and throughput concerning the limitations of the IoT resource. Preeti Chandrakar et al. [35] recommended a safe platform intended for patients by introducing a cloud-based authentication protocol in monitoring systems of e-health care. The conveyed method was tested as efficient, economical, and suitable for applications in real-time by the performance assessment. Impersonation, man-in-the-middle, session-key security, patient anonymity, along with substitution attacks were the drawbacks that caused the failure of this methodology. Kristen N. Griggs et al. [36] facilitated secure analysis together with the management of medical sensors by designing a BC-based smart contract. A structure was formed wherein the sensors communicate with SC and it was built with the aid of the Ethereum protocol that utilized private BC. To carry out an SC that could be assessed based on personalized threshold values, this structure used a permission consortium-managed BC. Unlike any other existing methodologies, this method produced better outcomes. However, with relevance to the key management, the problem arises in broadcasting, because the transactions of various smart devices had to wait at several nodes to authorize the subsequent block. Shin et al. [37] developed a secure framework for MQTT. They utilized Diffie–Helman and authenticated encryption for key management and encryption, respectively. The security level achieved by the protocol was acceptable, but it was computationally expensive. Mohsin et al. [38] proposed the IoTRiskAnalyzer framework which mostly focused on the physical security of IoMT without paying heed to the privacy of data. Seyed Morteza Pournaghi et al. [39] published a scheme called “MedSBA” based on BC technology along with attribute-based encryption, and it would store and register the medical data in a safe and well-organized manner. Medical content production, storing, and usage of PHI information were the three phases. To protect the patient’s privacy, attribute-based encryption combined with BC technology was utilized to record and store medical information. Even for the genuine user, the data were not easy to ingress the information during a decisive period of decision-making due to robustly encrypted, authentic, and digitally signed data and it was the defect of this methodology. Most of the existing work does not provide a complete security solution. In order to overcome this problem, we have developed a novel security problem for IoT-enabled healthcare systems.

## 3. Proposed Methodology

**Figure 1:**
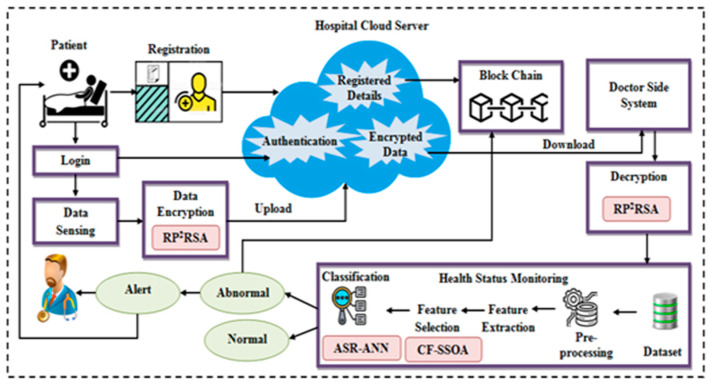
Structural design of the proposed framework.

The three layers on which IoT works are perception, network and application. Each layer of IoT have their own share of security problems. The physical layer deals with sensing data from surroundings. The network layer is responsible for routing that data to the IoT hubs. Lastly the application layer acts as interface between network and IoT devices. IoMT is basically a compilation of medical devices. Remote monitoring and processing the health-associated information devoid of going to the hospital is completed with the aid of IoMT. Next, the processed data are amassed in the CS for additional utilization. The IoMT devices collect a gamut of sensitive data and enable the physician and caretaker to make timely as well as data-driven decisions related to the patients’ HS. This device is fixed to the patients’ bodies. Because of third-party authentication and cloud-centered storage, the major consequence of the IoMT is sensitive healthcare data security, and privacy, together with storage management though it encompassed numerous advantages. The authentication and storage schemes used in IoMT are centralized in nature; hence, the prevailing security techniques in addition to privacy techniques are not suitable for IoMT devices. Therefore, the proposed work encompasses a BC-centered secure data storage system together with user authentication and HS prediction method. Initially, the user registration phase is performed. Then, during the login time, user authentication is performed. In the BC, the registered patient’s personal details are amassed, which renders user privacy. Data sensing together with data encryption using RP^2^RSA is made subsequent to authentication. Subsequently, the HS prediction system accepts the sensed data as the input. It envisages the normal as well as abnormal health conditions of patients and also transfers the alert message. For additional analysis, the abnormal data are amassed in the BC that aids the doctor to follow the record. For separating the normal and abnormal data, data collection, feature extraction (FE), and feature selection (FS), together with the classification process are carried out in the HS prediction system. The classification is completed utilizing the ASR-ANN technique. If the data are found to be abnormal an alert is sent to the patient. The dataset utilized consisted of 76 features and CF-SSOA was utilized for feature selection. The proposed technique’s structure is displayed in Figure 1.

### 3.1. Registration Phase

At first, the patient registers their personal details with the hospital’s cloud server. Once the registration is successful, the patient unique ID is generated. After that, the unique ID along with the timestamp (time at which the patient registers) is combined and hash coded using the Adler-32 hashing algorithm and forwarded to the patient along with their username and password. By using the hash code, the patient will be authenticated at the time of login. Adler-32 hashing is obtained by estimating two 16-bit checksums G, H and concatenating their bits into 32-bit output. G mentions the number of bytes and H is the sum of individual G values in each step. At the start, G, H are set to 1 and 0, respectively. The hash code generation process is demonstrated further.

❖ Let β be the patient unique ID and the timestamp of the patient at the registration time is represented as T. α is the string of bytes for which checksum is to be calculated. The patient ID and timestamp are combined together to convert them into hash codes. It is shown below.
  (1) α=βΤ
❖ Next, the hash code is generated for the combined values using the Adler-32 hashing technique which is mathematically expressed below. Here, 65521 represents the largest prime number smaller than 2^16^.
  (2) $$G=1+\alpha_{1}+\alpha_{2}+\ldots \ldots +\alpha_{n}\cdot \left|65521\right|$$
  where *n* denotes the length of α, Here, H is the sum of individual values of *G*.
  (3) $$H=\langle \left(1+\alpha_{1}\right)+\left(1+\alpha_{1}+\alpha_{2}\right)+\ldots +\left(1+\alpha_{2}+\ldots +\alpha_{n}\right)\rangle \cdot \left|65521\left.\right|\right.$$
❖ The hash code can be formed as follows,
  (4) $$Adl\left(\alpha \right)=\left(H\times 65521\right)+G$$

In Equation (4), Adl(α) shows the Adler-32 hash-coded value. This Hash code will be further sent to the patient and is used for authentication of the user at the login time and also for data encryption and decryption purpose. Once registration is completed, blockchain is enabled for that patient and their registration details are stored in the blockchain which provides privacy and security to ones’ details.

### 3.2. Blockchain

**Figure 2:**
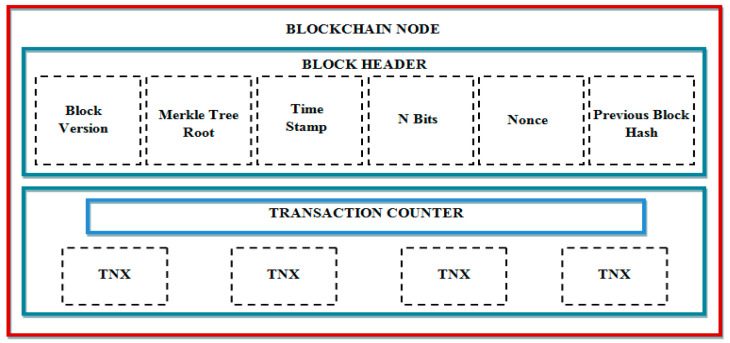
BC architecture.

Storing, sharing, and managing medical information together with patients’ privacy has been hard on account of the quick augmentation of the medical industry. The possible solution to share medical data securely was rendered by the BC for these issues. BC is a decentralized data structure. It renders unchangeable and irremovable transactions by means of generating a digital ledger. A chain of blocks is in the ledger that is cryptographically joined with one another. Once recorded on the BC ledger, blocks of data cannot be changed or removed. With the help of a linked list of encrypted transactions (it utilizes a hash instead of a pointer), the data in BC are primarily protected. In BC technology, the hash function can generate a hash value utilizing Secure Hash Algorithm 256 by means of encryption of inputted data. If an attacker tries to alter the data of a certain block, then the modified data and tampering will be visible. The proof of work (POW) helps to generate trustless consensus in the blockchain network. Modifying or corrupting a blockchain is practically impossible for a malicious attacker as it is computationally very expensive. Proof-of-work (POW) should be solved by the person who wants to add a new block to the BC network. The BC structure is exhibited in Figure 2.

‘4’ sections are there in the BC structure. The block size is rendered in the initial part, which is implied by bytes. Next, a block header is present for storing data, say BC version, HC of the previous block, as well as Merkle root. The root’s hash of the Merkle tree is termed the Merkle root. Merkle tree is basically a tree-like structure generated via all the transactions mentioned in the final section of the block. Timestamp, difficulty target (POW), along with a nonce (arbitrary variable for POW) are as well in the block header.

### 3.3. Login

The patients’ login phase is performed following the registration process. Here, the username, password, and unique patient ID, together with the secret HC are entered by the user for verification. User authentication occurs once the details are entered.

### 3.4. User Authentication

In this phase, comparisons of the details that are entered during the login and registration take place. The user will be authenticated if both match and also the data will be sensed. An error message will occur if not matched and also the data will not be sensed until authentication happens. Subsequent to successful patient authentication, the wireless body sensors (WBS) commence sensing the patients’ health data. Human life has become more suitable. Nevertheless, personal privacy is not guaranteed by IoT devices when exchanging data via wireless communication. Thus, data encryption is needed amid data exchange.

### 3.5. Data Encryption Using RP^2^-RSA

RP2-RSA algorithm encrypts the WBS data. Generally, Rivest–Shamir–Adleman (RSA) protects encrypted data utilizing a pair of keys. It is basically an asymmetric encryption algorithm. Key generation, encryption, together with decryption is the ‘3’ phases. Nevertheless, RSA is intensive computationally with extremely large integer numbers. Strong primes are vital in ensuring security. Thus, in the proposed work, the patients’ public, in addition to private keys will be connected and reversed and the HC generated as of Equation (4) will be added for rendering added security. This alteration in the top-notch RSA is called RP^2^-RSA. The mathematical model of the RP^2^-RSA is illustrated as,

#### 3.5.1. Key Generation

❖ At first, ‘2′ large prime numbers A,B are randomly chosen and gauge P=A∗B
❖ Next, the Euler totient function $\varphi \left(P\right)$ is gauged as,
  (5) $$\varphi \left(P\right)=\left(A-1\right)*\left(B-1\right)$$
❖ Choose a random integer r centered on the subsequent condition, $\gcd\left(r.\varphi \left(P\right)=1\right)$ and $1<r<\varphi \left(n\right)$ and calculate,
  (6) $$w=r^{-1}\cdot \mathrm{mod}\langle \varphi \left(P\right)\rangle$$
  wherein w signifies the multiplication inverse element of $\varphi \left(P\right)$ that fulfills the Equation (7),
  (7) $$r*w=1\cdot \mathrm{mod}\langle \varphi \left(P\right)\rangle$$
  Consequently, $\left(r,P\right)$ implies the public key and $\left(w,P\right)$ signifies the private key.
❖ Next, these public keys are joined with the private keys and reversed to make the secret key for ameliorated security. The secret key $\left(\delta \right)$ is generated utilizing the subsequent equation,
  (8) $$\delta =rw=>wr+Adl\left(\alpha \right)$$
  Additionally, this secret key is employed in the encryption as well as decryption of the information.

#### 3.5.2. Encryption

The data are encrypted as,

(9) $$\Omega =Μ\cdot \log\left(\delta \right)*\mathrm{mod}\left(P\right)$$

In Equation (9), Μ implies the original body sensor data and Ω signifies the encrypted data.

#### 3.5.3. Decryption

Centered on the equation below, the encrypted information is decrypted in the doctor system,

(10) $$Μ=\Omega \cdot \log\left(\delta \right)*\mathrm{mod}\left(P\right)$$

Like this, via the cloud server, the patient data are securely encrypted and transferred and decrypted at the doctor system. Next, HS prediction is performed on these data.

### 3.6. Health Status Prediction

Here, centered on the values attained as of the real-time WBS, the patient’s HS is predicted. Initially, the publicly available datasets are collected on the internet wherein the training is performed. After collecting the dataset, pre-processing is performed.

#### 3.6.1. Pre-Processing

Here, the unprocessed data are converted into a reasonable format for augmenting the accuracy along with the efficiency of the HS prediction system. A large redundant data and also missing values are present in the input dataset. Redundant data removal and missing value imputation are used as the pre-processing steps for processing them efficiently. The procedure of removing the same data that occurred manifold times on the dataset is termed redundant data removal. The procedure of replacing missing data on the input dataset with substituted values is termed the Missing value replacement. The values are added centered on the basic imputation method (mean) when values are missed in the over ‘1’ feature.

#### 3.6.2. Feature Extraction

After pre-processing, to ameliorate the classification’s accuracy in addition to time, the features are extracted from the pre-processed data. The total features in the dataset are lessened by the FE. It encompasses only the summarization of the utmost helpful information as of the actual set. The extracted features $\left(s_{i}\right)$ are expressed as,

(11) $$s_{i}=s_{1},s_{2},\ldots \ldots ,s_{n}$$

In Equation (11), n implies the number of features extracted as of the input dataset.

#### 3.6.3. Feature Selection Using CF-SSOA

Subsequent to FE, the FS process is established. It stands as the procedure of lessening the total input variables for developing the predictive model. Unimportant variables are eliminated and the classification’s performance together with accuracy is enhanced. In the proposed work, the correlation factor-induced salp swarms optimization algorithm (CF-SSOA) chose the essential features. It resolves intricate multi-optimization issues. It is basically a meta-heuristic population-centered nature-inspired algorithm. Salps are marine organisms, and it has jelly like tissues with the barrel-shaped body. For searching for food and better sustainability, they form a spiral-like chain. It comprises a leader and a follower. The leader will be at the front of the chain to guide the followers, whilst followers follow one another and revise their positions consequently. Nevertheless, local optima are brought about by the diversity between salps. This weakens the exploration capability of the algorithm. Premature convergence is also brought by the loss of a degree of dispersion amongst salps, which affects the optimization ability. For adjusting the leader and follower positions amid the food-searching process, correlation factors $\left(\tau^{1},\tau^{2}\right)$ are introduced for overcoming the aforesaid issues. The algorithm is renamed CF-SSOA because of the modifications made in the conventional SSOA. The CF-SSOA is elucidated further down.

**Step 1:** Let the initial swarm population be stated as $\left\{s_{i}=s_{1},s_{2},\ldots \ldots s_{n}\right\}$ (extracted features) in a d-dimensional search space. The salp swarms’ target is signified by the food source $\left(\Gamma \right)$. Initially, centered on the fitness calculation, the leader salp is chosen.

**Step 2:** Every Salp’s fitness $f\left(s_{i}\right)$ in the Salp population $s_{i}$ is computed via the closest distance betwixt the salp and food source. The fittest salp is chosen as the group’s leader. The followers are the remaining ones in the group. In searching for the target (food source), the leader salp guides the whole populace.

**Step 3:** Next, the position of the leader as well as the followers closest to the target is updated utilizing the equation below,

(12) $$s_{i}^{L}=\left\{\begin{array}{l} \Gamma +\xi^{1}\langle \left(u_{i}-l_{i}\right)\xi^{2}+l_{i}\rangle /\tau^{1}\;\xi^{3}\geq 0\\ \Gamma -\xi^{1}\langle \left(u_{i}-l_{i}\right)\xi^{2}+l_{i}\rangle /\tau^{1}\;\xi^{3}<0 \end{array}\right.$$

wherein $s_{i}^{L}$ signifies the leaders’ position on the *d*-th dimension, $\xi^{1}$ implies the adjustment parameter utilized for balancing the exploration and exploitation process, $\xi^{2}$ signifies an arbitrary number (algorithm coefficient) that controls the moving action, $\xi^{3}$ is also an arbitrary number that is employed to choose whether the leaders moving direction is closer or farther as of the food location, $u_{i},l_{i}$ signifies the upper and lower bounds of *d*-th dimension utilized to limit the leader as of exceeding the search space.

**Step 4:** Centered on the subsequent formula, the correlation factor is estimated

(13) $$\tau^{1},\tau^{2}=\frac{\sum_{i=1}^{n}s_{i}-\hat{s}}{\sqrt{\sum_{i=1}^{n}{\left(s_{i}-\hat{s}\right)}^{2}}}$$

Here, $s_{i}$ signifies the Salp population’s characteristics, $\hat{s}$ implies the mean values of the populace.

**Step 5:** The adjustment parameter $\xi^{1}$ is used for balancing the salps’ exploration as well as exploitation, which is gauged as,

(14) $$\xi^{1}=2\cdot \exp{\left(-\frac{4y}{Y}\right)}^{2}$$

In the above-given equation, y implies the present iteration and Y implies the maximal iterations.

**Step 6:** Next, for updating the followers’ position, Newton’s law of motion is employed, which is illustrated mathematically below,

(15) $$s_{i}^{F}=\frac{\frac{1}{2}\gamma \cdot t^{2}+I^{0}\cdot t}{\tau^{2}}$$

wherein $s_{i}^{F}$ implies the position of i−th follower on the d-dimensional search space, t signifies the response time, $I^{0}$ implies the initial speed of salps, γ implies the acceleration of the follower’s movement, which is

(16) $$\gamma =\frac{I^{\max}}{I^{0}}$$

The follower’s speed $\left(I\right)$ is evaluated additionally,

(17) I=s−s0t

The Equation (17) can well be modified as,

(18) siF=12siF+siF−1τ2

Here, $s_{i}^{F-1}$ signifies the ${\left(f-1\right)}^{th}$ follower Salp on the *d*-th dimension. The followers may attain an improved position (optimal solution) analogized to the current position by following the leader. Replacing the food source with a better position and updating the salp chain will continue the process. Lastly, the collection of optimal solutions (selected features $x_{j}$) is attained and modeled as,

(19) $$x_{j}=x_{1},x_{2},\ldots \ldots ,x_{N}$$

wherein N ascertains the number of selected features. Additionally, for envisaging the patient’s HS, these features are inputted into the classifier. The proposed CF-SSOA pseudocode is displayed in Algorithm 1.
**Algorithm 1:** Pseudocode for the proposed CF-SSOA**Input:** Extracted features $s_{i}$    **Output:** Selected feature $x_{j}$
    **Begin**    **Generate** initial swarm population    **Initialize** the food source Γ    **For** i=1:n           **Compute** the fitness $f\left(s_{i}\right)$ of each $s_{i}$           **Choose** the leader $s_{i}^{L}$ and followers $s_{i}^{F}$           **While** $\left(i<Y\right)$                        **Update**
$\xi^{1}=2\cdot \exp{\left(-\frac{4y}{Y}\right)}^{2}$                        **Calculate** the correlation factor $\tau^{1},\tau^{2}$                        **If**
i==1                                       **Renew the** position of leader                        **Else**                                       **Keep posted** the follower’s position                        **End if**                         **Improve** the salps based on upper and lower bounds            **End while**
    **End for**
    **Return** $x_{j}$
    **End**

#### 3.6.4. Classification via ANN

**Figure 3:**
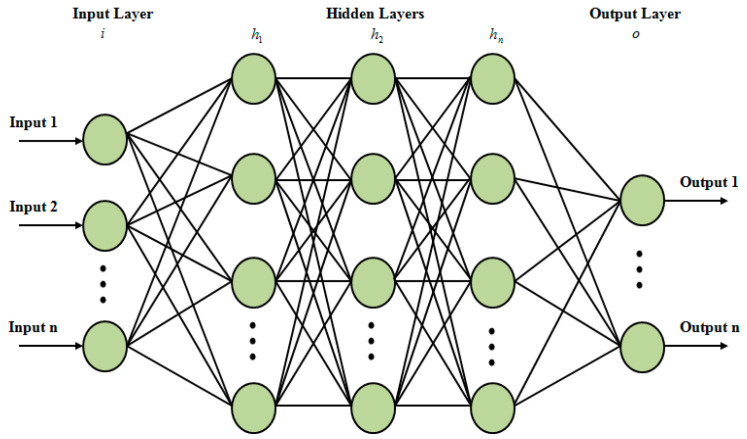
Baseline architecture of ANN.

Adapted SignReLU activation function-centered artificial neural network (ASR-ANN) takes care of the HS classification that classifies the status as normal and abnormal. The input layer (IL), hidden layer (HL), together with output layer are encompassed by the artificial neural network (ANN). The chosen features $x_{j}$ are inputted into the ANN’s IL. Subsequently, the random weights are initialized between the input-hidden and hidden-output layers. In the HL, for learning the intricate patterns in the inputted data, the sigmoid activation function (AF) is employed. Nevertheless, the sigmoid AF encompasses the issue of vanishing gradient, and also the random weight initialization brings about slower convergence. In the proposed work, in place of the sigmoid AF, the SignReLU AF is utilized for overcoming these issues. The advanced initialization technique initialized the weight values. The vanishing gradient issue is reduced and the slower convergence rate was avoided because of these modifications completed in the usual ANN. Such modifications made in the usual ANN are the so-called ASR-ANN. The baseline ANN architecture is exhibited in Figure 3.

The steps comprised in the classification process of ASR-ANN is detailed as,

**Step 1:** To commence the classification process, the chosen features $x_{j}$ are inputted to the SR-ANN’s IL and move towards the HL. In the HL, each input feature is multiplied with the input-hidden weight value $\left(w_{IH}\right)$. The hidden layer’s output $\left(h_{l}\right)$ is written as.

(20) $$h_{l}=\sum_{j=1}^{N}x_{j}\cdot w_{IH}+B_{H}$$

In the above-said equation, $B_{H}$ signifies the bias value of the hidden layer H. The advanced initialization method that is employed for the weight initialization is estimated as,

(21) $$w=\sqrt{\frac{2}{M^{l-1}+M^{l}}}$$

In the above-said equation, *M* implies the total neurons in $l^{th}$ and ${\left(l-1\right)}^{th}$ input-hidden and hidden-output layers.

**Step 2:** The SignReLU AF that is utilized in the HL is rendered by,

(22) $$\phi \left(h_{l}\right)=\left\{\begin{array}{l} h_{l}\;h_{l}\geq 0\\ a\cdot \frac{h_{l}}{\left|h_{l}\right|+1}\;h_{l}<0 \end{array}\right.$$

Here, $h_{l}$ implies the input of the nonlinear AF ϕ and a exemplifies the variable super parameter.

**Step 3:** Next, the last output in the output layer $\left(O\right)$ of the SR-ANN is computed utilizing the equation below,

(23) $$O_{l}=\sum_{j=1}^{M}\phi \left(h_{l}\right)\cdot w_{HO}+B_{O}$$

wherein $B_{O}$ implies the output layer bias value, $w_{HO}$ exhibits the hidden-output weight value that is initialized utilizing the Equation (21).

**Step 4:** Next, the cross-entropy loss function method evaluated the error value,

(24) $$Ε=-\frac{1}{N}\sum_{j=1}^{N}Y_{j}\cdot \log\left(Z_{j}\right)+\left(1-Y_{j}\right)\cdot \log\left(Z_{j}\right)$$

wherein $Y_{j}$ signifies the target value and $Z_{j}$ implies the classifier output.

The error values attained via the proposed classifier are low by the SignReLU AF as well as the advanced weight initialization methods. The classifier produces the output as normal and abnormal. The patient is regarded to be in good health condition and no medications are necessary if the output is normal. If the output becomes abnormal, it implies that the health condition of the patient is changed, and the patient requires medications for maintaining their health. Therefore, regarding the patient’s health, an alert message is sent to the doctor, patient, together with the corresponding caretaker. For future analysis, the health record is formed by securely storing the abnormal statuses and the abnormal parameter values (blood pressure, temperature, heart rate, etc.), along with the medication rendered by the doctor in the corresponding patients’ BC.

## 4. Result and Discussion

Here, the proposed framework’s experimental outputs have been contrasted with the outcomes of the prevailing techniques. We have utilized the dataset known as the Hungarian and Statlog data set which is available in the public domain. From the open source, the training dataset is gathered, and they are employed for the health status monitoring phase. The proposed technique has been executed with the assistance of JAVA. Based on certain performance criteria, the outcomes are compared and comprehensively listed below.

### 4.1. Performance Evaluation of RP^2^RSA

By analyzing the outcome metrics such as encryption time (ET), decryption time (DT), and key generation time, the execution of the encryption in addition to the decryption technique of the proposed data is verified. With the support of the prevailing techniques says Rivest–Shamir–Adleman (RSA), elliptic curve cryptography (ECC), and Elagamal, the proposed outputs are compared. The pictorial demonstration of the ET values as stated below.

**Figure 4:**
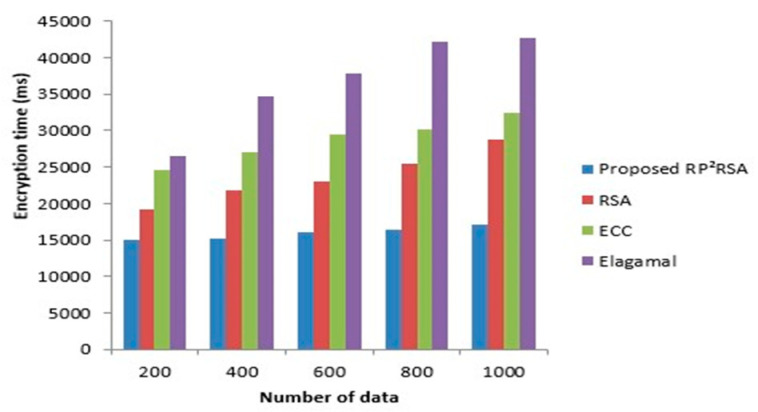
ET comparison of proposed and existing cryptographic method.

**Figure 5:**
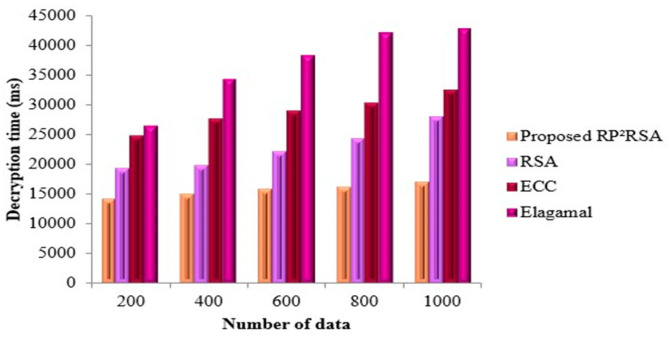
Performance assessment of proposed RP^2^RSA by means of DT.

**Figure 6:**
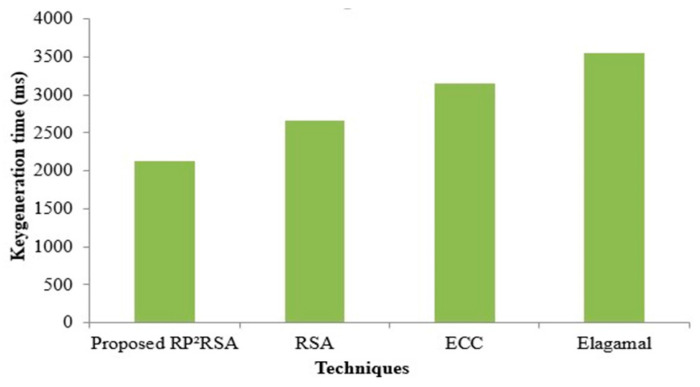
Key generation time of the proposed RP^2^RSA and existing RSA, ECC, and Elagamal.

ET is the time consumed to encrypt the quantity of data sensed by the sensor. If the ET is less, then it indicates good performance. In the RP^2^RSA methodology, the data on the WBS is encrypted to transmit the data safely. The time consumed to encrypt the data by the RP^2^RSA method is exhibited in Figure 4. Compared with the number of data Vs ET, the representation of the graph is made, which states that the augmentation in the data size increases the ET. Thus, in the RP^2^RSA methodology, the ET is 14,986 ms if the total data are less (200). The ET is 17,042 ms if there is a maximum of data (1000). However, the ET of the prevailing RSA is 19,124 ms for 200 numbers of data and this value is high when analogized to the RP^2^RSA technique. Similarly, for the other prevailing methodologies, there is a variation in the ET. The RP^2^RSA technique encrypts the data with a lesser amount of time than the other techniques which are evident in the graph. From Figure 5. a comparison of decryption time can well be seen clearly. The time consumed to decrypt the data after encryption is measured by the DT. A good performance is implied by less decryption time, the same as ET. By altering the number of data, the DT of the RP^2^RSA is given as 14,121 ms (200), 14,998 ms (400), 15,763 ms (600), 16,034 ms (800), and 16,874 ms (1000), in Figure 6. In contrast, 24,761 ms (200), 27,532 ms (400), 28,873 ms (600), 30,302 ms (800), and 32,442 ms (1000) are the DT of the prevailing ECC. The DT of the RP^2^RSA technique is lesser than the DT of the prevailing ECC. Likewise, there is a deviation in the prevailing RSA and Elagamal’s decryption time. Thus, in comparison with the other prevailing methods, the RP^2^RSA methodology is superior. 

**Figure 7:**
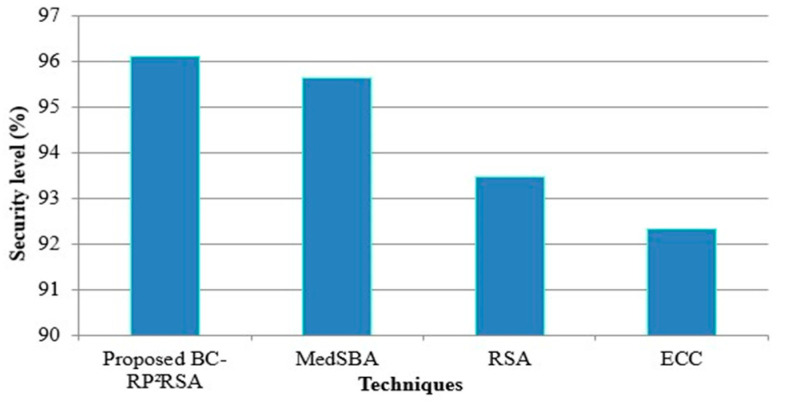
SL evaluation of proposed BC- RP^2^RSA technique.

The key generation time is stated as the period taken to generate the public key together with the private key for the user is illustrated as the key generation time. In comparison with the prevailing method, the proposed RP^2^RSA displays less time (2124 ms), and is revealed in Figure 6. On the contrary, to generate keys, the prevailing RSA needs 2662 ms, and the existent ECC and Elagamal take 3154 ms and 3548 ms, respectively. However, the only minimum time is taken by the RP^2^RSA technique. Therefore, by correlating with the prevailing method, the RP^2^RSA’s performance is better. From Figure 7. a comparison of key generation time can well be seen clearly.

Subsequently, the acquired security level (SL) for the BC- RP^2^RSA is evaluated. The SL acquired by the BC- RP^2^RSA is correlated with the prevailing MedSBA (medical data sharing centered on blockchain technology), RSA, and ECC in Figure 7. The BC- RP2RSA method accomplishes 96.123% of greater security which is proven by the evaluation. However, relatively, the prevailing system has a lower SL. Therefore, BC technology together with data encryption techniques is employed for data security and data encryption techniques. Thus, regarding the SL, the proposed structure has achieved superior performance.

### 4.2. Superiority Measure of the Proposed Classifier

The ASR-ANN classifier’s performance is utilized to classify the patient’s health status, and also it is proved by correlating the outcomes with the prevailing ANN, convolutional neural network (CNN), recurrent neural network (RNN), and deep belief neural network (DBNN). With regard to the accuracy, precision, recall, f-score, sensitivity, specificity, execution time, and memory usage (MU), the outcome is verified. Further, the validations of the performance are described.

In Table 1, the accuracy, precision, f-score, sensitivity, specificity, and recall of the ASR-ANN and existing methods are examined. The prevailing ANN acquires an accuracy of 95.152%, precision of 94.762% along with 95.387% of recall, which is lesser than the ASR-ANN methodology. Similarly, the accuracy of 93.383%, 91.725%, and 89.892%, the precision of 93.621%, 92.406%, and 91.01%, F-score of 93.985%, 92.359%, and 90.989% for the prevailing CNN, RNN, and DBN are achieved. Yet, higher performance like 95.893% of accuracy, 95.654% of precision, and 96.012% of f-score is obtained by the ASR-ANN proposed. Table 1 reveals the deviation in the estimation metrics such as sensitivity, and specificity, in addition to recall. The ASR-ANN classifier has greater sensitivity of 96.146%, specificity of 95.959%, and recall of 96.098%. It is apparent from the work, that the ASR-ANN classifier’s performance is better than the prevailing techniques.

**Figure 8:**
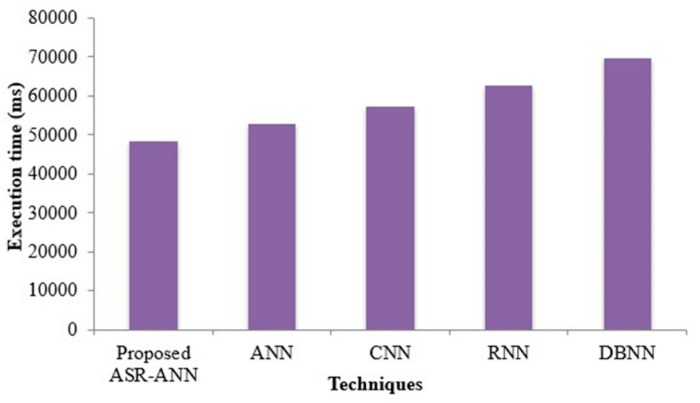
Execution time comparison of proposed and existing methods.

The ASR-ANN methodology and the existent classification’s performance regarding the ET are exhibited in Figure 8. The execution time of prevailing ANN, CNN RNN, and DBNN are 52,961 ms, 57,378 ms, 62,815 ms, and 69,721 ms, while the proposed technique’s execution time is 48,432 ms. By comparing with the prevailing techniques, the proposed ASR-ANN produces lesser execution time and exhibits good performance. The table below illustrates the MU.

The MU for the ASR-ANN and prevailing methodologies is tabularized in Table 2. The quantity of memory required in a system for processing the data is entitled as MU. Table 2 states that the ASR-ANN requires a memory of 165,432 kb. Analogized to the memory requirement of the prevailing ANN (1,989,791 kb), CNN (213,490 kb), RNN (246,701 kb), and DBNN (278,932 kb), the memory requirement is very low for the ASR-ANN. Thus, it is evident that the ASR-ANN has superior MU.

## 5. Conclusions

Here, a BC-centered secure data storage system is proposed together with user authentication and an HS prediction system. User registration, login, authentication, BC, data encryption, and HS monitoring are the subsequent processes present in the proposed framework. For providing security for the patient’s medical data, BC technology is employed. For predicting the patient’s health condition, an HS monitoring system is employed. Data encryption utilizing RP^2^RSA is proposed for securely transferring the patient IoT data in the CS. By contrasting the outcomes with some baseline techniques, the performances are estimated. The 96.123% SL with fewer times 48,432 ms is attained by the proposed work. The proposed model’s classification accuracy, and precision, together with the recall are 95.893%, 95.654%, as well as 96.098% correspondingly. Additionally, it needs less memory of 165,432 kb. It is apparent from the overall analysis as well as discussions, analogized to the top-notch methodologies that the proposed work performs better with higher security and accuracy and executes in a lesser time. In the future, the work will be ameliorated regarding the data load along with network failures utilizing advanced deep learning techniques. Furthermore, we would intend to utilize the proposed framework in other application scenarios of IoT.

## Figures and Tables

**Table 1:** Sensitivity, specificity, and recall of proposed and existing methods.

Techniques	Sensitivity (%)	Specificity (%)	Recall (%)
Proposed ASR-ANN	96.146	95.959	96.098
ANN	95.531	95.089	95.595
CNN	94.982	94.375	94.121
RNN	94.019	93.602	93.843
DBNN	93.158	92.428	92.204

**Table 2:** MU of the proposed and existing techniques.

Techniques/Performance Metric	Memory Usage (kb)
Proposed ASR-ANN	165,432
ANN	198,791
CNN	213,490
RNN	246,701
DBNN	278,932

## Abbreviations

RP^2^-RSA.	Reversed public-private keys combined Rivest–Shamir–Adleman
CF-SSOA	Correlation factor induced salp swarm optimization algorithm
ASR-ANN	Adapted SignReLU activation function centered artificial neural network
IoMT	Internet of Medical Things
BC	Blockchain
HS	Health Status
PID	Patient ID
DID	Device ID
ANN	Artificial neural network
CNN	Convolutional neural network
RNN	Recurrent neural network
DBNN	Deep belief neural network
